# Digital Engagement and Sleep Dysregulation in Young Adults: A Narrative Review

**DOI:** 10.7759/cureus.105030

**Published:** 2026-03-11

**Authors:** Muhammad Dawood Khan, Sameeha Junaidi, Mohammed I Dalbah

**Affiliations:** 1 General Medicine, Emirates Health Services (EHS) Hospitals, Ras Al Khaimah, ARE; 2 Pediatrics, Saqr Hospital, Ras Al Khaimah, ARE

**Keywords:** bedtime procrastination, problematic smartphone use, screen time, sleep quality, smartphone, young adults

## Abstract

Digital technology is embedded in daily life, and growing evidence suggests that evening and bedtime screen engagement may disrupt sleep in young adults. Associations appear stronger for bedtime exposure and problematic or addiction-like patterns of use than for total screen time alone. This review aims to synthesize current evidence on the relationship between smartphone and social media use and sleep outcomes in young adults, with particular attention to the timing of device use, problematic digital engagement, and psychological or behavioral factors that may influence sleep. A narrative literature review was conducted using a broad, non-systematic search of PubMed, Scopus, Web of Science, and Google Scholar. Observational, longitudinal, experimental, or interventional studies and higher-level syntheses were included when they contributed to conceptual understanding of the screen-sleep relationship. Evidence was organized thematically across domains, including overall associations, bedtime and in-bed use, problematic and addictive behaviors, bedtime procrastination, psychological mediators (rumination and fear of missing out), chronotype, objective exposure and sleep measures, content versus duration, and cross-cultural consistency. Across diverse populations and study designs, digital media use was consistently associated with poorer sleep outcomes, including reduced sleep quality, delayed sleep onset, shorter sleep duration, and daytime dysfunction. Bedtime and nighttime use showed stronger associations than general daily exposure, supporting sleep displacement and circadian disruption as key pathways. Problematic smartphone and social media use demonstrated particularly robust links with poor sleep and insomnia-related symptoms, and longitudinal evidence suggested bidirectional relationships between problematic phone use, impaired sleep, and depressive symptoms. Bedtime procrastination emerged as a central behavioral mediator, while rumination and fear of missing out contributed to cognitive-emotional arousal that sustains sleep disruption. Objective smartphone tracking and wearable sleep measures generally supported associations between nighttime phone activity and worse sleep, although short-term panel studies assessing brief exposure windows reported largely null effects. Experimental studies of short-term social media abstinence and structured digital detox interventions showed improvements in sleep quality and psychological well-being. Evidence indicates that sleep dysregulation in young adults is most strongly linked to bedtime screen exposure and problematic or compulsive digital engagement rather than screen time alone. Digital sleep hygiene strategies that reduce in-bed use and address problematic smartphone behaviors may improve sleep and well-being. Future research should prioritize longitudinal and intervention designs, clearer exposure definitions capturing habitual bedtime behaviors, and greater use of objective tracking to strengthen causal inference.

## Introduction and background

Digital technology has become inseparable from modern life, with smartphones and other screen-based media now shaping how people communicate, learn, work, and unwind. Alongside these benefits, growing synthesis-level evidence has raised concern that pervasive digital engagement, particularly during evening and bedtime hours, may compromise sleep health. Meta-analytic and systematic review findings across diverse countries and large samples consistently link higher electronic media use with poorer sleep quality, shorter sleep duration, and delayed sleep onset, with often stronger associations observed for problematic or addiction-like patterns than for time-based exposure alone [1,2]. Collectively, these data position digital engagement as a clinically relevant and potentially modifiable behavioral determinant of sleep dysregulation in young adults.

Young adults, particularly university and medical students, appear uniquely vulnerable to sleep disruption due to academic pressure, irregular schedules, and high evening digital engagement. Synthesis-level evidence specific to late adolescence and young adulthood indicates that higher digital media use is consistently associated with shorter sleep duration, poorer sleep quality, and longer sleep onset latency, with bedtime and nighttime use showing the strongest and most reproducible associations [2]. Consistent with this broader pattern, observational studies in medical and university student cohorts report that problematic or excessive smartphone use is linked to worse sleep quality, commonly reflected in higher Pittsburgh Sleep Quality Index (PSQI) scores, and to sleep-relevant impairments such as longer sleep latency, reduced sleep duration, and daytime dysfunction [3-5]. Together, these findings support the view that sleep dysregulation in young adult students is closely intertwined with both the timing and behavioral intensity of smartphone and social media engagement, rather than screen exposure alone [2-5].

Medical students are a high-risk subgroup for sleep disturbance given intensive academic workload, clinical responsibilities, and elevated stress. Observational evidence consistently links problematic smartphone use with poorer sleep quality (higher PSQI) and with sleep-domain impairments, including longer sleep latency, reduced sleep duration, and greater daytime dysfunction; downstream functional impacts, including poorer academic performance, have also been reported in student cohorts [3-5]. A medical student-focused systematic review and meta-analysis further confirmed that this association is moderate, statistically significant, and consistent across diverse settings, supporting problematic smartphone use as a potentially modifiable behavioral factor in sleep dysregulation [6].

Importantly, the association between smartphone use and sleep disturbance extends beyond academic settings. In community-based adult samples, excessive smartphone use is associated with poorer sleep quality and greater psychological distress, including higher symptoms of depression, anxiety, and stress, suggesting that these relationships are relevant at the population level [7]. In workplace settings, bedtime smartphone use has been linked to reduced sleep quality and to downstream occupational consequences, such as poorer work performance and increased interpersonal conflict, with sleep quality functioning as an intermediary pathway in these relationships [8]. Similarly, among healthcare workers, where shift schedules and occupational stress already elevate vulnerability to sleep disruption, problematic smartphone use is highly prevalent and is significantly associated with poor sleep quality, underscoring the clinical and occupational relevance of digital overuse [9].

Multiple complementary mechanisms may explain how digital engagement disrupts sleep. Physiologically, short-wavelength (“blue”) light emitted from smartphone screens can suppress nocturnal melatonin secretion, delay circadian timing, and impair sleep initiation. Experimental evidence supports this pathway: in a pre-post intervention study of university students, the use of a blue light-reducing filter during evening smartphone use was associated with a significant improvement in subjective sleep quality, reflected by a reduction in global PSQI scores from 7.63 ± 2.10 to 5.37 ± 1.89 (p < 0.001), alongside improvements in sleep latency and related sleep components [10].

From a behavioral perspective, smartphone use may disrupt sleep by delaying bedtime and promoting bedtime procrastination, particularly through habitual pre-sleep engagement and nighttime checking behaviors. In student and community samples, these patterns are associated with poorer sleep quality and sleep-domain impairment, most consistently longer sleep latency, shorter sleep duration, and greater daytime dysfunction, supporting time displacement as a key pathway linking problematic smartphone use to sleep disturbance [5,11].

Psychological and cognitive processes further shape the screen-sleep relationship. In young adults, sleep disturbance is tightly coupled with mental health: during the COVID-19 pandemic, poorer sleep quality correlated strongly with higher depressive symptoms, anxiety, and perceived stress, even though screen time itself was not significantly associated with PSQI scores in that sample [12]. Consistent with this broader sleep-mental health linkage, community-based data show that excessive smartphone use co-occurs with poorer sleep quality and greater psychological distress [7]. Extending this pattern mechanistically within student populations, mediation modeling among university students suggests that sleep disturbances may partially explain the association between mobile phone addiction and depressive symptoms, with gender differences also reported [13].

Emerging mediation research suggests that the link between problematic smartphone engagement and sleep impairment is partly explained by behavioral delay and cognitive-emotional arousal. In a general adult sample, bedtime procrastination partially mediated the association between problematic smartphone use and poorer sleep quality, consistent with time displacement as a proximal pathway to sleep disruption [11]. Complementing this behavioral mechanism, data from college students indicate that fear of missing out and rumination function as psychological mediators, both independently and sequentially, linking mobile phone addiction to poorer sleep quality, supporting the role of pre-sleep cognitive and affective activation in sustaining sleep disturbance [14]. Collectively, these findings suggest that sleep disruption is not solely attributable to screen exposure per se but also to the motivational and affective processes that prolong engagement and delay disengagement at night [11,14].

Smartphone use and sleep quality also appear embedded within broader lifestyle and self-regulatory patterns. In a large college-student sample, higher physical activity was associated with better sleep quality, and structural modeling suggested that this relationship operates partly through greater self-control and lower mobile phone addiction, including a significant sequential pathway from physical activity to self-control to reduced phone addiction to improved sleep [15]. These findings position problematic smartphone use as a potentially modifiable mediator within a wider behavioral framework, supporting a multidimensional model in which sleep health reflects interactions among lifestyle behaviors, self-regulatory capacity, and technology-related habits [15].

Although the literature linking screen exposure to sleep outcomes is extensive, it remains methodologically heterogeneous across study populations, exposure definitions (e.g., duration-based versus problematic use), sleep measurement approaches, and the explanatory models used to interpret findings. While systematic reviews and meta-analyses offer valuable synthesis and pooled estimates, they typically concentrate on particular age groups or media-use constructs and therefore may not fully integrate the behavioral, psychological, and physiological pathways implicated in sleep disruption into a single coherent framework [1,2,6]. For this reason, a narrative synthesis is well-suited to contextualize and connect findings across study designs and conceptual perspectives, with the goal of producing an integrated interpretation of how digital engagement contributes to sleep dysregulation.

This narrative review aims to synthesize contemporary evidence on the relationship between screen time, particularly smartphone use, and sleep outcomes, with emphasis on behavioral patterns, psychological mediators, physiological mechanisms, and functional implications. By integrating observational findings, mediation analyses, experimental research, and higher-level syntheses, this review seeks to provide a comprehensive and clinically relevant overview of how digital engagement influences sleep health across diverse populations.

## Review

Methodology

Study Design and Conceptual Framework

This study was conducted as a narrative literature review synthesizing contemporary evidence on the relationship between screen exposure, particularly smartphone and social media engagement, and sleep-related outcomes. A narrative approach was selected to support conceptual integration across heterogeneous study designs, exposure definitions, and sleep measurement frameworks, and to allow interpretative linkage of behavioral, psychological, and physiological mechanisms that may connect digital engagement with sleep dysregulation. The objective was not to generate an exhaustive systematic inventory of all available studies, but to develop an evidence-informed narrative that highlights consistent patterns, methodological considerations, and emerging explanatory models across the field.

Scope of the Review

The review primarily focused on young adults, typically aged 18 to 30 years, with particular emphasis on university and healthcare student populations, given their high digital engagement and well-documented vulnerability to sleep disruption. However, evidence from broader adult, occupational, and population-based samples was also considered when it directly informed mechanistic interpretation, functional implications, or the generalizability of findings beyond academic settings. Screen-related exposures included total screen time, evening or bedtime use, smartphone use duration, nocturnal checking behaviors, problematic smartphone use, mobile phone addiction or involvement, and problematic social media engagement. Sleep outcomes were the primary endpoint and included sleep quality, sleep duration, sleep onset latency, insomnia symptoms, sleep disturbances, sleep efficiency, and daytime dysfunction. Psychological and contextual factors, including stress, anxiety, depression, fear of missing out, and rumination, were incorporated when examined as mediators, moderators, or closely linked correlates within the screen sleep pathway.

Literature Identification Strategy

A broad, non-systematic search was conducted in PubMed, Scopus, Web of Science, and Google Scholar to identify empirical studies and higher-level syntheses addressing screen exposure and sleep outcomes. Search terms were developed around two conceptual domains, digital engagement and sleep-related parameters, and included combinations of screen time, smartphone use, bedtime phone use, nighttime screen use, problematic smartphone use, mobile phone addiction, social media use, sleep quality, sleep disturbance, sleep duration, insomnia, sleep onset latency, bedtime procrastination, fear of missing out, and rumination. Boolean operators were used to combine exposure and outcome terms, and database-specific syntax was adapted to indexing conventions. Reference lists of relevant publications were hand searched to identify additional studies that contributed substantively to the conceptual aims of the review.

Study Selection

Studies were considered eligible if they examined a screen-related exposure and reported at least one measurable sleep outcome among young adults or student populations. In addition, population-level, occupational, or mechanistic studies were included when they provided evidence directly relevant to the relationship between screen use and sleep. We included observational research (cross-sectional and longitudinal designs), experimental or interventional studies, and high-quality narrative reviews, systematic reviews, and meta-analyses when these sources strengthened inference or advanced explanatory understanding.

Eligible studies were published between 2019 and 2026, with the final search completed on February 20, 2026. Only studies published in English were included. Studies were excluded if they did not measure sleep outcomes, focused solely on psychological variables without any sleep assessment, enrolled exclusively pediatric or older adult samples without relevant subgroup analyses, or lacked sufficient methodological clarity to allow meaningful interpretation. Following initial selection, duplicate records were removed. Study inclusion was determined primarily by relevance to the review aims, methodological rigor, and contribution to conceptual and mechanistic synthesis, rather than by formal systematic review procedures.

Data Extraction and Narrative Synthesis

Key study characteristics were extracted to support structured comparison across designs and populations. Extracted elements included authorship, year, country, study design, sample characteristics, exposure measurement methods, sleep assessment instruments, analytic approach, and principal findings. Given substantial heterogeneity in exposure definitions, measurement tools, and statistical reporting, quantitative pooling was not pursued. Findings were synthesized narratively and organized into thematic domains aligned with the structure of the review, including overall associations between digital engagement and sleep outcomes, timing and bedtime-related exposure patterns, problematic and addictive digital behaviors, behavioral and cognitive emotional mediators such as bedtime procrastination and psychological arousal, objective and physiological measurement evidence, distinctions between screen duration and content type, and regional or cultural consistency of observed associations. Interpretation prioritized consistency across studies, theoretical coherence, and the strength of inference offered by longitudinal, experimental, and objectively measured evidence, while acknowledging the predominance of cross-sectional designs in the broader literature.

Narrative Findings

The evidence was synthesized across the following seven thematic domains: overall associations between digital media use and sleep outcomes; timing of exposure and in-bed use; problematic and addictive patterns of engagement; behavioral mediators, including bedtime procrastination; psychological mediators, including rumination and fear of missing out; objective and physiological evidence; and regional and cross-cultural consistency. This narrative synthesis highlights convergence across observational, longitudinal, and experimental studies while noting areas of heterogeneity and remaining gaps.

Discussion

Overall Association Between Digital Media Use and Sleep Outcomes

Across observational, longitudinal, and experimental studies, digital media use is consistently associated with poorer sleep outcomes, including reduced sleep quality, delayed sleep onset, shorter sleep duration, and increased daytime dysfunction [16-20].

Importantly, evidence also suggests that timing of exposure matters: population-based analyses distinguishing temporal patterns of screen use indicate that evening/bedtime screen time shows stronger associations with impaired sleep quality than more general exposure [21]. These associations have also been documented across student, healthcare, and general young adult populations, suggesting that sleep disruption related to screen exposure is a widespread phenomenon rather than a context-specific finding [22,23].

Large population-based data further demonstrate dose-response relationships between social media engagement and sleep disturbance, reinforcing the public health relevance of this association [24]. Finally, lifespan-oriented syntheses and consensus work indicate that these associations are not confined to a single age group, with broadly similar patterns observed across adolescents, young adults, and adults, and with timing and behavioral engagement repeatedly emerging as key correlates [25,26].

Timing of Screen Exposure and Bedtime Use

A recurring and robust finding is that nighttime and pre-sleep phone use shows a stronger association with sleep disruption than total daily exposure; studies that separate overall use from bedtime behaviors report that pre-sleep use and nocturnal checking are more closely linked to later bedtimes, shorter sleep duration, and poorer sleep quality, whereas total daily use shows weaker or less specific associations [21,27].

University-based and young adult studies consistently show that greater screen exposure, particularly electronic device use in the hour before bedtime, is associated with poorer subjective sleep, including higher PSQI scores, longer sleep latency, and more frequent sleep disturbances, with several datasets also suggesting a dose-response pattern as exposure increases [18,19,28]. These patterns are also evident in healthcare trainee and medical student samples: among young adult healthcare students, higher recreational screen time is associated with poorer sleep quality and related outcomes [22], and multinational data in medical students similarly link nighttime screen use and, importantly, smartphone addiction symptoms to worse sleep quality, suggesting that both timing and compulsive use patterns may exacerbate an already stress-vulnerable group [29].

Large national survey data in university students show that screen use after going to bed is associated with more insomnia symptoms and meaningfully shorter sleep duration, consistent with sleep displacement as a key pathway [30]. Parallel evidence from adult samples similarly links greater (especially nighttime) screen exposure with shorter sleep duration, longer sleep latency, and more nocturnal awakenings, supporting both displacement and circadian-related disruption as plausible mechanisms [31].

Problematic and Addictive Patterns of Digital Use

Beyond exposure duration, dysregulated and addictive patterns of digital engagement show particularly strong links to sleep disturbance. In student and young adult samples, problematic mobile phone involvement and smartphone addiction are consistently associated with poorer sleep quality, insomnia-related symptoms, and daytime dysfunction [17,23]. Similar associations are observed for problematic social networking site use, which correlates with worse PSQI-defined sleep quality and broader psychological distress [32]. Among nursing students, smartphone addiction is accompanied by a very high burden of poor sleep quality [33].

Longitudinal evidence strengthens this association. In a three-wave, cross-lagged panel analysis, problematic mobile phone use predicted subsequent depressive symptoms, with significant indirect effects operating through bedtime procrastination and poorer sleep quality, including a sequential (chain) pathway [16]. Bidirectional effects were also observed, depressive symptoms predicted later problematic phone use, and sleep quality showed reciprocal associations with depression, supporting a potentially self-reinforcing cycle linking sleep disturbance, problematic digital behavior, and affective symptoms over time [16].

Experimental and intervention studies further support this relationship. Short-term social media abstinence and structured digital detox interventions have been associated with improvements in sleep quality and psychological well-being, suggesting that problematic digital behaviors are modifiable and may be meaningfully implicated in sleep health [34,35].

Psychological and Behavioral Mediators

Several studies converge on bedtime procrastination as a key behavioral mechanism linking problematic digital engagement to sleep disruption. Longitudinal evidence suggests that problematic mobile phone use can precede later psychological burden through a cascading pathway involving bedtime procrastination and deteriorating sleep quality, with reciprocal effects indicating a potentially self-reinforcing cycle over time [16]. Complementing this, pandemic-era data indicate that psychological stress reactions may increase bedtime procrastination partly by intensifying smartphone addiction symptoms, highlighting how contextual stress can amplify maladaptive digital habits that delay sleep onset [16,36].

Cognitive and emotional mechanisms also contribute to sleep disruption. In a large mediation study of Chinese college students, rumination and fear of missing out were significant mediators of the association between mobile phone addiction and poorer sleep quality, including evidence for a sequential pathway in which addiction increases fear of missing out, which increases rumination, and subsequently worsens sleep outcomes [14]. Chronotype may further shape vulnerability: longitudinal evidence indicates that eveningness predicts poorer sleep quality and greater insomnia symptoms, which, in turn, mediate downstream risk for problematic social media use, psychological distress, and daytime sleepiness, suggesting that late chronotypes may be particularly susceptible to reinforcing cycles of late-night engagement and sleep impairment [37].

Objective and Physiological Evidence

Although much of the literature relies on self-reported screen exposure and sleep outcomes, objective assessment increasingly supports the association between nighttime smartphone engagement and sleep disruption. In a population-based study using high-resolution tracking data, objectively measured nighttime smartphone activity was associated with shorter sleep duration and poorer sleep quality, strengthening inference by reducing exposure misclassification and recall bias [38]. In addition, small observational work combining subjective sleep measures with app-based sleep tracking suggests that prolonged nighttime smartphone use is accompanied by poorer sleep quality and more fragmented sleep patterns, including lower sleep efficiency and increased wake episodes [39].

At the same time, evidence from short-term prospective panel designs is more equivocal. In a repeated-measures study that paired real-life markers of phone use with wearable sleep tracking across multiple days, brief exposure windows were largely not associated with sleep duration or sleep efficiency [40]. This contrast may indicate that sustained and habitual nighttime use, rather than isolated short-term exposure, is more relevant to persistent sleep disruption, and that study duration and the temporal definition of exposure can substantially influence observed effects [38-40].

Screen Type, Content, and Duration

Evidence increasingly suggests that bedtime screen duration is more strongly linked to adverse sleep outcomes than the specific content consumed. In a large national survey of Norwegian university students, screen use after going to bed was associated with shorter sleep duration and higher odds of insomnia symptoms, and these associations did not differ meaningfully between social media and other screen-based activities, supporting a primarily time-dependent rather than content-specific effect [30].

In parallel, data from medical student populations indicate that behavioral dependence adds explanatory value beyond exposure time, with smartphone addiction symptoms independently predicting poorer sleep quality even after accounting for general nighttime screen use, suggesting that compulsive use patterns may amplify vulnerability in high-stress groups [29].

Regional and Cultural Evidence

Regional studies from South Asia consistently document a high prevalence of extended screen exposure alongside poor sleep quality in student and young adult samples, with higher daily or bedtime phone use associated with longer sleep latency, shorter sleep duration, and more frequent night awakenings [19,20,27,41,42]. In parallel, population-level evidence from Western settings shows that heavier social media engagement, particularly greater frequency and intensity of use, is associated with higher odds of sleep disturbance, supporting cross-cultural consistency in observed risk patterns [24]. An India-focused narrative synthesis further consolidated these findings by concluding that increased digital screen exposure, especially during evening and nighttime hours, is consistently linked to impaired sleep outcomes across Indian young adult populations, reinforcing the generalizability of the association beyond single-institution samples [26].

Limitations

This narrative review was intended to integrate concepts and mechanistic explanations across heterogeneous study designs rather than to provide an exhaustive, protocol-driven systematic appraisal. Accordingly, the evidence base remains methodologically diverse, with substantial variability in how screen-related exposures are defined (e.g., overall screen time, nighttime use, device type, content, or problematic use) and how sleep outcomes are measured (subjective questionnaires versus objective indices). A major constraint is the predominance of cross-sectional studies, which limits causal inference and increases vulnerability to reverse causation. Across studies, reliance on self-reported screen use and sleep outcomes may introduce recall bias, social desirability effects, and exposure misclassification. Residual confounding is also likely, given incomplete adjustment for key determinants of sleep (e.g., baseline mental health symptoms, chronotype, caffeine and stimulant use, academic workload, physical activity, and concurrent media multitasking). Publication bias cannot be excluded, and the literature may disproportionately represent university student samples, potentially limiting generalizability to non-student young adults and other occupational or cultural contexts.

Implications, recommendations, and future directions

The consistent association between bedtime digital engagement and adverse sleep outcomes supports practical interventions that prioritize timing and behavioral regulation rather than focusing only on total daily screen exposure. Clinically and in university settings, education on digital sleep hygiene may be useful, particularly emphasizing reduced in-bed smartphone use, minimizing nighttime checking behaviors, establishing a technology-free wind-down period, and reducing evening light exposure through brightness reduction or blue-light filtering when screen use is unavoidable. Screening for problematic smartphone use in high-risk groups such as medical students may help identify individuals who could benefit from targeted behavioral support.

Future research should strengthen causal inference by expanding longitudinal and intervention-based studies, with clearer temporal definitions of exposure that capture habitual bedtime behaviors. Greater use of objective measures, including passive smartphone tracking and wearable sleep metrics, would reduce measurement error and clarify which patterns of use most strongly predict sleep disruption. Studies should also examine moderators such as chronotype, stress, mental health symptoms, and cultural context, and should assess clinically meaningful outcomes, including academic performance, occupational functioning, and mental health trajectories.

## Conclusions

This narrative review demonstrates a consistent association between digital media use and adverse sleep outcomes across populations and study designs. The relationship is strongest for nighttime use, bedtime exposure, and problematic or addictive digital behaviors, rather than total screen time alone. Experimental and intervention studies show that temporary abstinence from social media and short-term digital detox programs are associated with improved sleep quality and improved well-being outcomes, supporting the potential causal relevance of digital engagement to sleep disruption. These findings support the promotion of digital sleep hygiene, with particular emphasis on limiting bedtime screen exposure and addressing problematic smartphone use. Future research should prioritize longitudinal and intervention-based designs, integrate objective exposure measures, and further examine developmental and cultural moderators.
